# The Therapeutic Potential of the Essential Oil of *Thymbra capitata* (L.) Cav., *Origanum dictamnus* L. and *Salvia fruticosa* Mill. And a Case of Plant-Based Pharmaceutical Development

**DOI:** 10.3389/fphar.2020.522213

**Published:** 2020-11-24

**Authors:** S. A. Pirintsos, M. Bariotakis, M. Kampa, G. Sourvinos, C. Lionis, E. Castanas

**Affiliations:** ^1^ Department of Biology, University of Crete, Heraklion, Greece; ^2^ Botanical Garden, University of Crete, Rethymnon, Greece; ^3^ Laboratory of Experimental Endocrinology, School of Medicine, University of Crete, Heraklion, Greece; ^4^ Laboratory of Clinical Virology, School of Medicine, University of Crete, Heraklion, Greece; ^5^ Clinic of Social and Family Medicine, School of Medicine, University of Crete, Heraklion, Greece

**Keywords:** traditional medicine, Southeastern Europe, Mediterranean, Near East, synergy, regulatory affairs, clinical trials, antiviral

## Abstract

This review performs a comprehensive assessment of the therapeutic potential of three native herbs of Crete (*Thymbra capitata* (L.) Cav., *Salvia fruticosa* Mill. and *Origanum dictamnus* L.), their phytochemical constituents, health benefits and issues relevant to their safety, within a translational context. Issues discussed comprise: 1) Ethnopharmacological uses of the three herbs, reviewed through an extensive search of the literature; 2) Systematic analysis of the major phytochemical constituents of each plant, and their medicinal properties; 3) To what extent could the existing medicinal properties be combined and produce an additive or synergistic effect; 4) Possible safety issues. We conclude with a specific example of the use of a combination of the essential oils of these plants as an effective anti-viral product and the experience gained in a case of a plant-based pharmaceutical development, by presenting the major steps and the continuum of the translational chain.

## Introduction

Plants are traditionally used, in different forms, for the treatment of diseases, since the Neolithic Era (Hardy, 2019). Different populations used native flora for the production of preparations, efficient in curing different diseases and conditions (Fabricant and Farnsworth, 2001). The Cretan area (KK, Crete and Karpathos floristic region) is such an example, as its evolutionary history preserved a very diverse flora, with more than 2000 indigenous species, including a high number of endemics (Dimopoulos et al., 2013). Cretan people abundantly use plants and greens in different aspects of their life. Cretan diet, a specific entity of the Mediterranean diet, uses a diversity of plants for culinary purposes (Renaud, 1995 and references herein), while a variety of plants are used as decoctions and infusions for recreational or medical purposes. We systematically analyzed the habits of a rural population in Crete and reported that use of different plant infusions, alone or in combination, resulted in a significant protection from common cold and influenza infections (Lionis et al., 1998). Based on this study, we have focused on three of the most efficient plants (*Coridothymus capitatus*, *Salvia fruticosa* and *Origanum dictamnus*) and developed a supplement, efficient at combatting upper respiratory tract infections (Duikler et al., 2015; Anastasaki et al., 2017). 

In this review, we analyze in depth the health benefits of these plants, their most prominent active compounds and their combination. In addition, we review our experience with the development of a nutraceutical product, discussing potential bottlenecks encountered during the process.

## The Plants

### Taxonomy and Distribution

According to the Euro + Med plantbase, ***Coridothymus capitatus* (L.) Reichenb. fil**. is a dwarf-shrub. It is the only member of the monospecific genus *Coridothymus*. According to the Euro + Med plantbase (Accessed at June 2017), the name *Coridothymus capitatus* (L.) Reichenb. fil. is synonym of *Thymbra capitata* (L.) Cav., which is included in Kingdom–Plantae, Division–Tracheophyta, Subdivision–Spermatophytina, Class–Magnoliopsida, Superorder–Asteranae, Order–Lamiales, Family–Lamiaceae Lindl., Genus–*Thymbra* L.

*Accepted name:* *Thymbra capitata* (L.) Cav., Homotypic synonyms: *Coridothymus capitatus* (L.) Rchb. f., *Origanum capitatum* (L.) Kuntze, *Satureja capitata* L., *Thymus capitatus* (L.) Hoffmanns. & Link.

***Salvia fruticosa* Mill**. is also a member of the Labiatae family. The taxon is included in Kingdom–Plantae, Division–Tracheophyta, Subdivision–Spermatophytina, Class–Magnoliopsida, Superorder–Asteranae, Order–Lamiales, Family–Lamiaceae Lindl., Genus–*Salvia* L.

*Accepted name*: *Salvia fruticosa* Mill, Heterotypic synonyms: *Salvia baccifera* Etl., *Salvia clusii* Jacq., *Salvia cypria* Unger & Kotschy, *Salvia fruticosa* subsp. *cypria* (Unger & Kotschy) Holmboe, *Salvia fruticosa* subsp. *thomasii* (Lacaita) Brullo and al., *Salvia incarnata* Etl., *Salvia libanotica* Boiss. & Gaill., *Salvia lobryana* Azn., *Salvia marrubioides* Vahl, *Salvia ovata* F. Dietr., *Salvia sipylea* Lam., *Salvia subtriloba* Schrank, *Salvia sypilea* Lam., *Salvia thomasii* Lacaita, *Salvia triloba* L. f., *Salvia triloba* subsp. *calpeana* (Dautez & Debeaux) P. Silva, *Salvia triloba* var. *calpeana* Dautez & Debeaux, *Salvia triloba* subsp. *libanotica* (Boiss. & Gaill.) Holmboe, *Sclarea triloba*(L. f.) Raf.

***Origanum dictamnus* L.** is a local endemic of Crete, member of Labiatae family. The taxon is included in Kingdom–Plantae, Division–Tracheophyta, Subdivision–Spermatophytina, Class–Magnoliopsida, Superorder–Asteranae, Order–Lamiales, Family–Lamiaceae Lindl., Genus–*Origanum* L.

*Accepted name*: *Origanum dictamnus* L., Heterotypic synonyms: *Amaracus dictamnus* (L.) Benth., *Majorana dictamnus* (L.) Kostel., Heterotypic synonyms: *Amaracus tomentosus* Moench, *Origanum dictamnifolium* St.-Lag., *Origanum saxatile* Salisb.

*Coridothymus capitatus* is found throughout the Mediterranean region distributed in all Mediterranean countries with the exception of France. *Salvia fruticosa* is distributed in Italy, Sicily, and the eastern Balkans, including Cyprus, while *Origanum dictamnus* is local endemic of Crete (Fielding and Turland, 2005).

## Methodology

**FIGURE 1:**
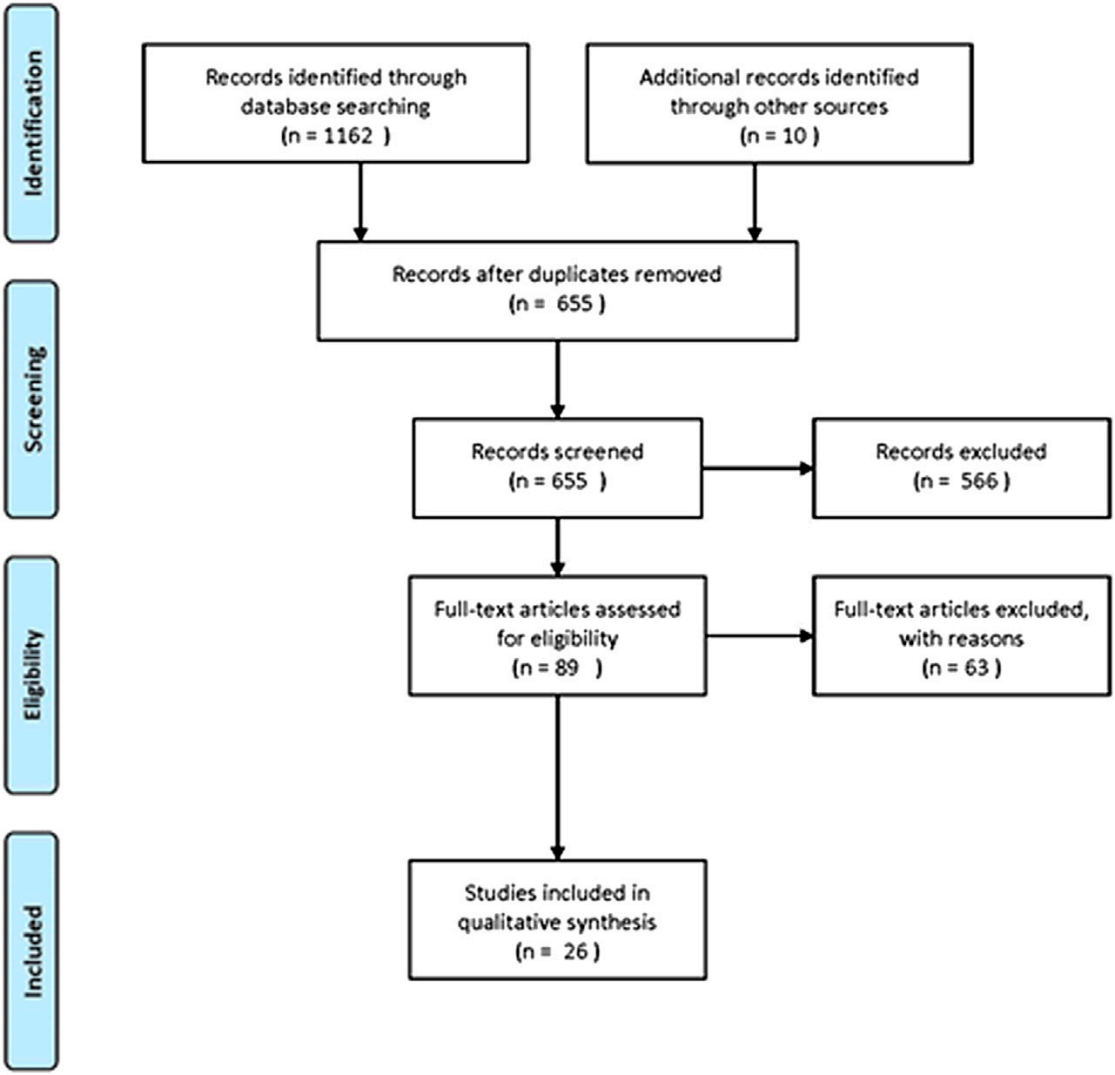
PRISMA flow diagram for the ethnobotanical and ethnopharmacological data.

In the context of this review, PubMed, Scopus and Google Scholar were used for the retrieval of the relevant bibliography. The following inclusion criteria were used for the systematic review of the three plants: articles published in English, articles concerning countries of the Mediterranean basin and also including Jordan, Syria, Iran and Iraq, as well as studies with the following keywords in their text: *Coridothymus capitatus*, *Salvia fruticosa* and *Origanum dictamnus*. The PRISMA flow diagram, presenting the results of our search, is depicted in Figure 1. The primary search identified 1,162 articles, plus 10 articles from other sources. After the implementation of the eligibility criteria, 26 articles were finally incorporated. Any evaluation of studies concerning the plant quality was out of scope and has not been included in this review.

Concerning the synergistic interactions of the main constituents of the essential oil (see below) the review was carried out through a literature searching of the same databases using the following keywords in all fields: “synerg* AND (carvacrol OR eucalyptol OR 1,8-cineole OR beta-Caryophyllene).” The selection of manuscripts was based on the following criteria: articles published in English and articles where only documented synergy between pure substances was reported. The primary search identified 4,101 articles, with 1,538 from PubMed, 1,910 from Google Scholar and 653 from Scopus and after the implementation of the eligibility criteria, excluding the repetitions, 25 articles were finally included in the qualitative analysis.

## Ethnopharmacological Uses of the Plants

*Coridothymus capitatus*, *Origanum dictamnus* and *Salvia fruticosa* are renown since antiquity for their pharmaceutical properties (Karousou et al., 1998). The first two are included in the Dioscorides book “*De Materia Medica*.” *Salvia fruticosa* was introduced in the Iberian Peninsula for cultivation by Greeks and Phoenicians and elements of these cultivations can be discovered today in several parts of the Iberian coast (Rivera et al., 1994). *O. dictamnus* is also mentioned from the Minoan era, through the centuries from several physicians and philosophers including Asklepios, Euripides, Aristotle, Hippocrates, Theophrastus, Virgil and Galen (Vrachnakis, 2003).

*Coridothymus capitatus* is known in local cultures with several common names among which Spanish oregano, thyme, headed thyme, conehead thyme, agrio thymari (αγριο θυμάρι), thymari (θυμάρι), tomilho de Creta and tomilho de Dioscórides, *Salvia fruticosa* is known with the names Greek sage and faskomilo (φασκόμηλο), while *Origanum dictamnus* is known as diktamo (δίκταμο), dittany of Crete and dictamo de Creta.

The ethnobotanical and ethnopharamacological studies concerning the medical use of *Coridothymus capitatus*, *Salvia fruticosa* and *Origanum dictamnus,* as well as from studies on local herbal markets in Eastern Mediterranean region, are presented in Tables 1–3 respectively, together with the plant part and their mode of preparation/use.

**TABLE 1:** Medicinal uses of *Coridothymus capitatus* derived from ethnobotanical and ethnopharmacological studies.

Area	Local name	Plant part	Medicinal use	Mode of preparation/use	Literature
Israel (Golan heights and west bank region)		Foliage	Heart diseases, paralysis, diabetes, track pain and inflammation and respiratory system	An infusion of one tsp foliage in a cup of water is made and taken 1/3 times/day until improvement occurs	Said et al. (2002)
Turkey (west Anatolia)	Kekik	Herbs	For cough colds, stomachache	Internally, infusion, tea, 3 days on empty stomach	Honda et al. (1996)
Jordan (northern Badia region)	Zater faresy	Leaves	Heart and respiratory diseases, diabetes, inflammation and used as rubefacient	Infusion	Alzweiri et al. (2011)
Pelestinian area	Thyme	—	Respiratory and urinary digestive systems and inflammation	—	Ali-Shtayeh et al. (2000)
Israel	—	—	Cardiovascular system and digestive system	—	Palevirtch et al. (1986)
The levant countries	Headed thyme	—	Internal diseases, eye diseases, stomach intestine	—	Lev (2002)
Jordan	Conehead thyme	Branch	Digestive system, respiratory system	—	Lev and Amar (2002)
Cyprus	Conehead thyme	Aerial parts	Respiratory tract diseases (catarrh and common cold)	—	Lardos (2006)
Jordan (Showbak)	Thyme	Aerial parts	Antispasmodic	Syrup	Al-Qura’n (2009)
Jordan	—	Leaves	Pectoral, stomachic, and to treat urinary tract infections	Infusion	Al-Khalil (1995)
Israel (northern Israel)	—	Leaves	Heart disorders. Swelling and drops. Indigestion. Local paralysis	Tisane. Taken as needed decoction. Used as a drink for 40 days. Steam-bath. Treatment is given for a duration of a month	Dafni et al. (1984)
Spain (Barros area, Badajoz province)	—	Leaves	Disestive, tonic, carminative	—	Vázquez et al. (1997)
Cyprus	Throumpin, agrio thymari	Flowering herb, flower, leaf	Respiratory tract	Tea, chew	Lardos and Heinrich (2013)
Turkey	Ballı kekik,bal kekiği, zahter, beyazkekik	Erial parts, flowering branches, esssential oil,, fixed oil	Diabetes, analgesic, Pharyngitis, cold, flu, pleasureand medicinal tea	Drink oneteacup2–3 times a dayfor3–4 weeks/apply 2–3 timesadayfor1–2 Weeks in, Exo,Lo, Lpw,Spc	Sargin (2015)
Egypt (Sinai peninsula)	Zaatar, Hasha	Flowering tops, leaves	Infusion as analgesic, sedative, digestive diseases, spasms, vomiting, flatulence. Liver diseases (jaundice)	—	Eissa et al. (2014)

**TABLE 2:** Medicinal uses of *Salvia fruticosa* derived from ethnobotanical and ethnopharamacological studies.

Area	Local name	Plant part	Medicinal use	Mode of preparation/use		Literature
Israel (golan heights and west bank region)		Foliage	Stomach ache, intestinal gas and inflammation, diabetes and sexual weakness	An infusion of 50 g in 1 L is prepared and taken orally, 150 cc, 1–3 times/day until improvement occurs		Said et al. (2002)
Turkey (west Anatolia)	Almiya çalbasi = almiya yaği	Herbs	For flatulence and constipation for babies. For colds, cough, stomachache	Internally, volatile oil is applied on nipples before nursing to alleviate the flatulence and constipation of a baby. Infusion, as herbal tea		Honda et al. (1996)
Greece	Faskomilo, elelisfakos	Erial parts	Hypotension, diabetes, laryngitis, pharyngitis, tonsillitis, constipation, diarrhea, spasmolytic, anemia, brain stimulant, calmative, depression, common cold, arthritis, hair loss, hair tonic, stomatitis, dysmenorrhoea, stimulant	Infusion, decoction, external application (footbath, message, washings)		Hanlidou et al. (2004)
Israel	Sage	Leaf	Hemorrhages, intestinal diseases and pains	—		Lev & Amar (2000)
Jordan	Sage	Leaf	Hemorrhages, intestinal diseases and pains	—		Lev & Amar (2002)
Pelestinian area	White sage	—	Digestive system, prostate disorders, skin disorders	—		Ali-Shtayeh et al. (2000)
Israel (northern Israel)	—	Leaves	Indigestion. Coughs and colds	Tisane. Taken as needed		Dafni et al. (1984)
Jordan (northern Badia region)	Meirameieh	Foliage	Stomachache, flatulence, inflammation, diabetes and sexual weakness	Decoction		Alzweiri et al. (2011)
Cyprus	Tree lobed sage	Leaf, tip of shoot	Febrile conditions (not specified), gastro-intestinal tract disorders (tenesmus), malaria fever, absent or delayed menstruation, repellent against arachnids, insects, snakes, respiratory tract diseases (catarrh and common cold, cough), supporting treatment in pleurisy and pneumonia, wounds	External (topical applications, baths), oral		Lardos (2006)
Jordan (Showbak)	Sage, clary	Leaves stems	Sedative, for wounds healing	Syrup		Al-Qura’n (2009)
Jordan	Meriamiah	—	Astrigent and antidandruff	Lotion		Al-Khalil (1995)
Jordan (Mujib nature reserve	Meriamiah, Miramieh	Leaves	Spasm, common cold, intestinal gases	Infusion (50 g in l lof water) is made and taken orally 3 times a day. Infusion is also prepared with tea by adding 3–5 g to a cup of tea		Hudaib et al. (2008)
Cyprus	Spatzia, faskomilo	—	Hypotension, diabetes, estrogen action, diarrhea, dyspepsia, spasmolytic, anti-aging, anti-perspirant, brain stimulant, depression, and nervous tonic, aphthae, gingivitis, dysmenorrhea, antiseptic, diuretic, tonic		Decoction, infusion/oral (potion), external (Compress, washings)	Karousou & Deirmentzoglou (2011)
Jordan (Ailoun heights region)	Meriamia, Meirameieh	Leaves	Antispasmodic	Decoction		Aburjai et al. (2007)
Palestinian area	White sage	Leaves	Anti inflammatory gargle, antiseptic, antitussive, antihaemorrhoids pain, antirheumatic, anti stomach, disturbances, astringent, carminative, hypotensive	—		Ali-Shtaueh et al. (1998)
Israel (northern Israel)	—	Leaves	Indigestion goughs and colds	Tisane is taken as needed		Dafni et al. (1984)
Turkey (Marmaris, Muğla)	Adaçayı, almakeyik, almageyik	Leaves	Stomachache, flatulence, cold, tonsillitis, laxative, antipyretic	Infusion, int		Gürdal and Kültür (2013)
Palestine/west bank	—	Leaves	Diarrhea	—		Jaradat et al. (2016)

**TABLE 3:** Medicinal uses of *Origanum dictamnus* derived from ethnobotanical and ethnopharmacological studies.

Area	Local name	Plant part	Medicinal use	Mode of preparation/use	References
Greece	Diktamos, erontas	Erial parts	Diabetes, liver disorders, spasmolytic, stomach ulcer, cholesterol, brain stimulant, headache, antiseptic, sanative, diuretic, dysmenorrhoea, antibacterial activities, aphrodisiac, stimulant	Infusion, external application (washings, compress)	Hanlidou et al. (2004)
Iran	Poneh kouhi	—	Dyspnea, bronchitis, allergy, depression, itch, dementia, abortifacient	Decoction	Naghibi et al. (2005)
Cyprus	Diktamo	Erial parts	Liver disorders, headache, neuralgia, cough, antiseptic, sanative, aphrodisiac, tonic	Infusion/oral (potion), external (compress)	Karousou and Deirmentzoglou (2011)
Europe	Dictamnus	—	Diabetes mellitus, obesity, disorders of lipoprotein metabolism and other lipidaemias, mood disorders, sexual dysfunction (not caused by organic disorder disease), epilepsy, acute nasopharyngitis (common cold), acute pharyngitis (unspecified), acute tonsillitis (unspecified), gingivitis and periodontal diseases, other specified disorders of teeth and supporting structures (toothache NOS), gastric ulcer, gastritis and duodenitis, diseases of liver, xerosis cutis, inflammatory polyarthropathies, pain in joint, acute nephritic syndrome, renal tubule-interstitial diseases, calculus of kidney and ureter, disorder of urinary system (unspecified), absent scanty and rare menstruation, pain and other conditions associated with female genital organs and menstrual cycle, dysmenorrhea unspecified, spontaneous abortion, long labor, cough, headache, convulsions (not elsewhere classified), multiple superficial injuries (unspecified), multiple open wounds (unspecified)	—	Martínez-Francés et al. (2015)

Based on this information, medicinal uses, in an ethnobotanical context, suggest that the three species have a highly antimicrobial and anti-inflammatory action. In addition, they are active against various targets and diseases, including diseases of the respiratory, digestive and urinary systems.

## Phytochemical Constituents

The essential oils of the three plants is produced from collected plant material, air dried in the dark, at room temperature (25°C) for 10 days. For analysis, after steam distillation, 1 ml of volatile oils were diluted with 2 ml of ether and filtered through anhydrous sodium sulfate to remove water traces and were stored at 4°C. Analysis was performed, as described previously (Duikler et al., 2015), by Gas Chromatography-Mass Spectroscopy (GC-MS, Shimadzu, QP 5050 A), with a MDN-5 column and a Quadrupole Mass Spectrometer as detector, after injection of 2 μL. The carrier gas was helium, the flow rate 0.9 ml/min. The sample was measured in a split mode procedure (1:35). For GS-MS detection an electron ionization system was used with ionization energy at 70 eV. The interested reader should refer to Duikler et al. (2015) for further analytical details.

**FIGURE 2:**
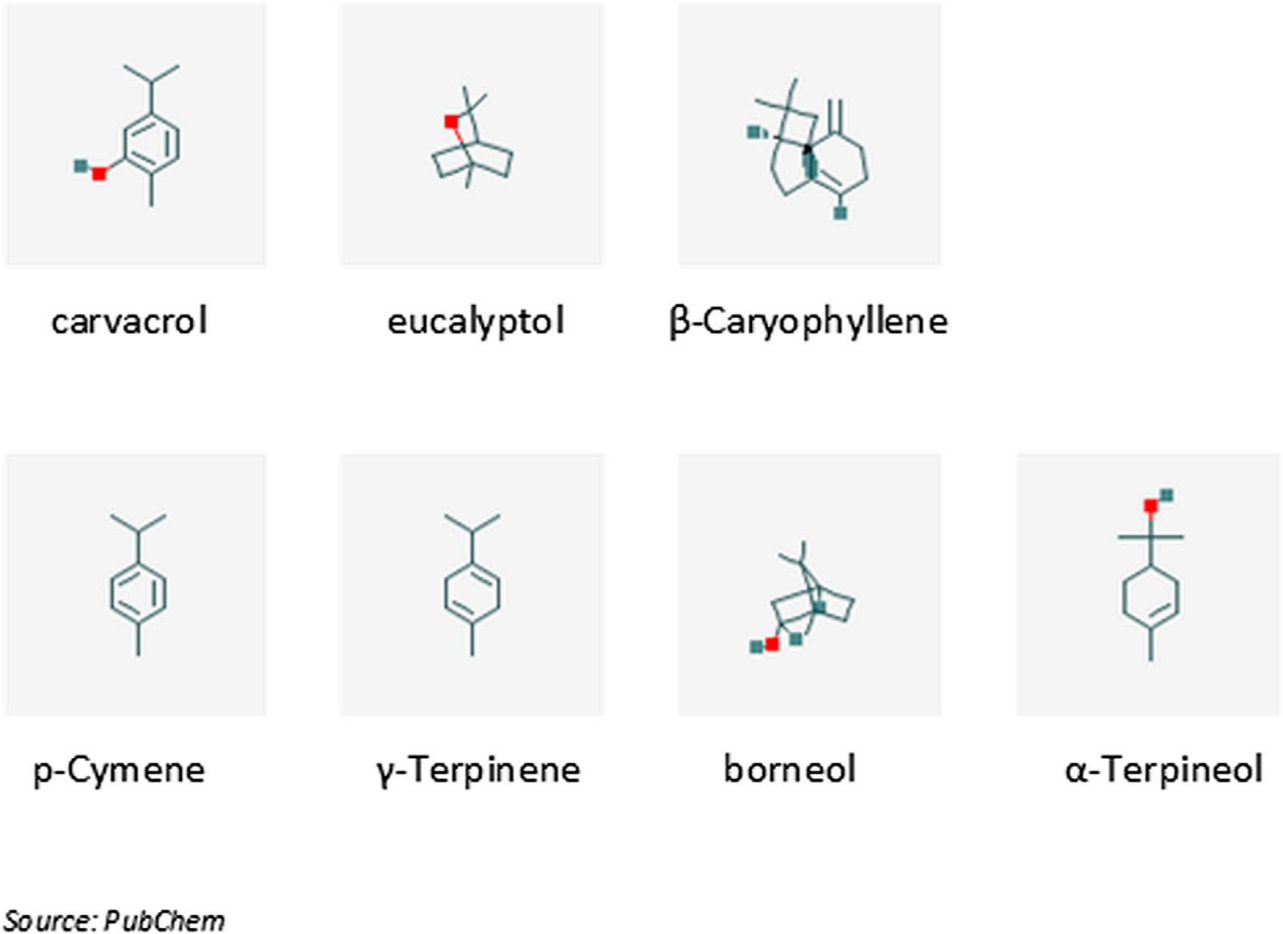
The main phytochemical constituents contained in the essential oil of plants discussed in this review. IUPAC names: 2-methyl-5-propan-2-ylphenol (carvacrol), 1,3,3-trimethyl-2-oxabicyclo [2.2.2] octane (eucalyptol), (1R,4E,9S)-4,11,11-trimethyl-8-methylidenebicyclo [7.2.0] undec-4-ene (β-Caryophyllene), 1-methyl-4-propan-2-ylbenzene (*p*-Cymene), 1-methyl-4-propan-2-ylcyclohexa-1,4-diene (γ-Terpinene), (1S,2R,4R)-1,7,7-trimethylbicyclo [2.2.1] heptan-2-ol (borneol), 2-(4-methylcyclohex-3-en-1-yl)propan-2-ol (α-Terpineol).

The chemical constituents of the essential oils of the three plants, as well as their combination (1.5% v/v of pure essential oils in olive oil carrier), used in the context of upper respiratory tract infections are presented in details as Supplemental Material in Duikler et al. (2015). According to this description (see also Figure 2) carvacrol (52.7%) is the main constituent of the mixture, followed by eucalyptol (12.77%) and *β*-caryophyllene (3.41%). The compounds *p*-cymene, *γ*-terpinene, borneol and *α*-terpineol participate with concentrations 1.32, 1.17, 1.68 and 1.06% respectively, while the rest 15 compounds participate with less than 1% (Figure 2).

Carvacrol (5-isopropyl-2-methylphenol) is an aromatic monoterpene with molecular weight 150.2 g/mol. It is a constituent of many essential oils, especially those found in plants of the family Lamiaceae, where the *Origanum vulgare* subsp. *hirtum* (Link) (Kokkini and Vokou, 1989).

Eucalyptol (1,8-cineole) is a cyclic ether monoterpene (molecular weight 154.3 g/mol), which is found as a constituent of plant essential oils (its name is derived from its presence in essential oils of *Eucalyptus globosus*). It is also considered as a major monoterpene emitted by vegetation into the atmosphere (Guenther et al., 1994).

β-caryophyllene is a natural bicyclic sesquiterpene with molecular weight 204.4 g/mol that is a constituent of many essential oils, such as clove or rosemary oil (Bernardes et al., 2010).

Concerning the other compounds, *p*-cymene is a naturally occurring organic compound that is characterized as a hydrocarbon related to a monoterpene. Its molecular weight is 134.2 g/mol and it is a constituent of several essential oils, such as the oil of cumin (Allahghadri et al., 2010). *γ*-terpinene is a terpene with molecular weight 136.3 g/mol. It is a major component of essential oils made from *Citrus* fruits (Lücker et al., 2002). Borneol is a bicyclic monoterpene with molecular weight 154.3 g/mol containing exactly two rings which are fused to each other. *α*-Terpineol is a naturally occurring monoterpene alcohol, derived from several sources, including oil of pines. Terpineol, due to its pleasant odor (similar to lilac), is used as an ingredient in perfumes, cosmetics, and flavors (Kim and Chung, 2000).

## Uses of Phytochemical Constituents

Apart from the ethnobotanical and ethnopharmacological uses of plants mentioned above, several uses of the aforementioned phytochemical constituents are also documented:

### Carvacrol

Carvacrol is the basic constituent of many essential oils, mainly of oregano species (Labiatae) (Kokkini and Vokou, 1989; Kirimer et al., 1995; Baydar et al., 2004; Stefanaki et al., 2016); it is characterized by its strong antioxidant properties (similar to those of vitamin E and ascorbic acid) (Mastelic et al., 2008). It is also known for its pronounced antimicrobial and antibacterial action (Suntres et al., 2015). The antimicrobial activity of several essential oils has been related to their content of carvacrol (García-Beltrán and Esteban, 2016), while, in a number of studies, the antimicrobial and the antibacterial action of the pure compound has also been investigated (Ben Arfa et al., 2006).

The antibacterial action of carvacrol extends to a variety of Gram-positive and negative bacteria (Kim et al., 1995; Rattanachaikunsopon and Phumkhachorn, 2010a; Ravishankar et al., 2010; Rivas et al., 2010). The compound acts as a transmembrane monovalent cation (hydroxyl proton for a potassium cation) exchanger (Suntres et al., 32,015). Therefore, in addition to its hydrophobic characteristics, allowing its accumulation in the membrane, the presence of free hydroxyls is essential for its antibacterial and antimicrobial activity (Ben Arfa et al., 2006).

An anti-inflammatory action of carvacrol is also documented (Landa et al., 2009; Hotta et al., 2010) and relies to the decreased production of inflammatory mediators, including cytokines, prostaglandins, enzymes, nitric oxide (NOS) and reactive oxygen species (ROS) (da Silva Lima et al., 2013). The authors suggest that carvacrol’s anti-inflammatory effects specifically induced IL-10 release, leading, subsequently, to the reduction of IL-1β and prostanoids production. In addition, it was reported that the inhibition of prostaglandin synthesis (Wagner et al., 1986) by carvacrol, is the basis of its antinociceptive activity. Additional mechanisms of antinociceptive actions of carvacrol include its agonistic activity for Transient Receptor Potential Vanilloid 3 (TRPV3), an ionic channel implicated in hyperalgesia and possibly skin sensitization (Xu et al., 2006). In addition, carvacrol activates and promptly desensitizes the specific sensor of environmental irritants TRPA1 ion channel/receptor (Xu et al., 2006).

### Eucalyptol (1,8-Cineole)

1,8-cineole (cineole), also known as eucalyptol, is the principal constituent of most *Eucalyptus* oil-preparations. It is employed in drug preparations, as a percutaneous enhancer and anti-inflammatory agent, decongestant and antitussive, while in aromatherapy it is used as a skin stimulant (Santos and Rao, 2000; Yuan et al., 2006).

Juergens et al. (2004), reported that 1,8-cineol inhibited Th1/Th2-associated cytokine production by human lymphocytes and monocytes. This anti-inflammatory action resulted in a subsequent inhibition of cytokine-induced airway mucus hypersecretion.

1,8 cineole is also known for its analgesic action (Guimarães et al., 2013). Santos and Rao (2000) suggested that the potent anti-inflammatory action of the compound was associated with an outstanding peripheral analgesic effect. They also suggested that 1,8- cineole-induced analgesic effects are not associated to neuronal toxicity, while Khalil et al. (2004) documented that 1,8-cineol, in a concentration-dependent manner, acted directly on sensory nerves and blocked nerve excitability (Lima-Accioly et al., 2006; Guimarães et al., 2013), through a direct activation of TRPM8 channels, which is a specific heat/cold receptor. TRPM8 activation in sensory nerves by 1,8-cineole, produced a specific analgesic effect, in cases of peripheral nerve injury (Proudfoot et al., 2006).

### β-Caryophyllene

β-caryophyllene is a widespread plant volatile compound, which is a selective ligand of the CB_2_ cannabinoid receptor, specifically related to analgesia and inflammation, and devoted of psychotropic actions, mediated by CB_1_ (Gertsch et al., 2008; Klauke et al., 2014). Therefore, CB_2_ receptor-selective agonists, like *β*-caryophyllene, are potential analgesic drug candidates, in various types of pain (such as neuropathic pain) (Guindon and Hohmann, 2008; Kinsey et al., 2010).

### *p*-Cymene

*p*-cymene is a precursor of carvacrol. It has an antimicrobial action *per se*, but it is less effective than carvacrol, when used alone (Kiskó and Roller, 2005), because *p-*cymene lacks a hydroxyl group, which is an important radical for the antimicrobial activity (Ultee et al., 1999; 2002). *p-*cymene also demonstrated an enhanced antinociceptive action in animal models of neurogenic and inflammatory pain (Guimarães et al., 2013).

### *γ*-Terpinene

The antimicrobial activity of *γ*-terpinene is debatable, with positive (Sato et al., 2007) and negative (Sivropoulou et al., 1996) reports, against strains of Gram-positive or Gram-negative bacteria.

### Borneol

Borneol is a known medicinal substance in Chinese and Indian traditional medicine. Borneol and its derivatives, possess antimicrobial (Corrêa et al., 2012), anti-inflammatory (Ehrnhöfer-Ressler et al., 2013) and antiviral activity. It also posesses a highly stimulatory action at GABA_A_ receptors (Granger et al., 2005; Manayi et al., 2016). GABA is the predominant inhibitory transmitter in the mammalian central nervous system and stimulation of GABA_A_ receptors produces anxiolysis, sedation, anesthesia and myorelaxation (Chebib and Johnston, 2000).

Borneol also expressed topical analgesic action. Wang et al. (2017), providing evidence for a topical analgesic efficacy in humans; TRPM8 was identified as its main molecular target. Moreover, borneol demonstrated anti-thrombotic effects, related to its anticoagulant properties (Li et al., 2008; Chen et al., 2015), possibly related to its potent modulation of the nitrite/nitrate reductase activity (Tang et al., 2009).

### *α*-Terpineol

α-terpineol is characterized by a moderate antibacterial activity, with a broad antibacterial spectrum. It is worth mentioning that *a*-terpineol showed antibacterial activities against penicillin-resistant bacterial strains (Kotan et al., 2007).

α-terpineol exerts central and peripheral antinociceptive activity (Quintans-Júnior et al., 2011). It also inhibits NF-κB and subsequently down-regulats the expression of proinflammatory IL-1β and IL-6 cytokines (Held et al., 2007; Hassan et al., 2010), mainly explaining the compound’s antinociceptive and anti-inflammatory properties (Oliveira et al., 2012).

α-terpineol also exhibited a gastroprotective action in ethanol-induced gastric ulcers (Souza et al., 2011) and beneficial effects in the cardiovascular system (Ribeiro et al., 2010). The proposed mechanism of action included endothelial NO-related vasorelaxation and activation of the NO–cGMP pathway.

## Registered Medical Applications

A series of clinical studies (most of them double-bind placebo-controlled trials, Table 4) evaluated the efficacy of 1,8-cineol (eucalyptol), in several medical issues. 1,8-cineol (eucalyptol) is a licensed medicinal product in Germany, since many years, in intestine-soluble capsules, for the treatment of acute and chronic bronchitis, sinusitis, respiratory infections and rheumatoid-like joint diseases (Juergens et al., 2003; Guimarães et al., 2013).

**TABLE 4:** Clinical studies concerning the evaluation of efficacy of the main constituents of essential oil preparation from *Thymbra capitata* (L.) Cav., *Salvia fruticosa* Mill. and *Origanum dictamnus* L., in several medical conditions.

Compound	Symptome	Clinical trial	References
1,8-cineol (eucalyptol)	Anti-inflammatory activity in bronchial asthma	Double-bind placebo-controlled trial	Juergens et al. (2003)
Cineole	Acute nonpurulent rhinosinusitis	Double-blind, randomized, placebo-controlled trial	Kehrl et al. (2004)
Cineole	Acute non-purulent rhinosinusitis	Prospective, randomised, double-blinded controlled study	Tesche et al. (2008)
Cineole (eucalyptole)	Chronic obstructive pulmonary disease	Placebo-controlled double-blind trial	Worth et al. (2009)
Cineole	Asthma	Placebo-controlled, double-blind trial	Worth and Dethlefsen (2012)
Cineole	Acute bronchitis	Placebo-controlled double-blind trial	Fischer and Dethlefsen (2013)
1,8-cineol (in eucalyptus oil)	Pain and inflammatory responses after total knee replacement	Randomized clinical trial	Jun et al. (2013)
1,8-Cineole	Preoperative anxiety	Randomized clinical trial	Kim et al. (2014)

Borneol and *p*-cymene have also been subjects of clinical studies for induced analgesia and treatment of the Fish Tapeworm disease respectively (Vartiainen, 1950; Wang et al., 2017).

## Synergy of Phytochemical Constituents

According to a classical definition, synergy occurs when the combined effect of two or more substances is greater than the sum of the individual agents (Breitinger, 2012). When the registered effect is an add up of individual actions, the action is reported as additive. Recently, the definition of synergy has been clarified from two points of view, the pharmacodynamic (enhanced therapeutic actions on the same target) and pharmacokinetic (no direct interaction but a multi-target behavior) (Yang et al., 2014; Zhou et al., 2016). The main reason of employing combinations of active substances with synergistic interactions is to reduce the administered amount of each compound and to increase the biological activity of a preparation/mixture against a specific target. In addition, this strategy diminishes the chance of pharmaceutical resistance of the pathogenic organism (Araújo et al., 2016).

Synergistic interactions were known for specific substances long time ago. For example, Barbaste et al. (2002) proposed a “cascade” reaction from lipophilic to hydrophilic antioxidants, enhancing their biological effects, while Vardar-Ünlü et al. (2003) suggested that the crude essential oil of *Thymus pectinatus* var *pectinatus* was more effective as antioxidant (DPPH assay) than its main active components (thymol and carvacrol). Testing the antioxidant activity of thymol, carvacrol and *p*-cymene, Milos and Makota (2012) showed synergistic effects in any combination of any two of the above compounds. Moreover, *p*-cymene, the precursor of carvacrol was found to enhance the bactericidal activity of carvacrol when used in combination (Kiskó and Roller, 2005; Rattanachaikunsopon and Phumkhachorn, 2010b). Synergistic effects of *p*-cymene have also been reported in relation to thymol and *γ*-terpinene (Dauqan and Abdullah, 2017).

Synergistic interactions of carvacrol, eucalyptol (1,8-cineole) and *β*-caryophyllene are presented in Table 5. Synergy was also reported between carvacrol and other minor constituents, such as, *p*-cymene, carvacrol and thymol. Eucalyptol also appeared to have synergistic effects with other constituent, including camphor, terpinene-4-ol and caryophyllene oxide. Additionally, synergy was also reported between minor constituents, such as between camphor and *p*-cymene.

**TABLE 5:** Synergistic interactions of carvacrol, eucalyptol and *β*-Caryophyllene. Synergistic interactions of minor constituents of the essential oil reported in the same articles are also reported.

Substances	Biological activity	Interaction	References
Thymol/carvacrol	Antigenotoxicity	Synergy	Quintero Ruiz et al. (2017)
Carvacrol/p-cymene	Antigenotoxicity	Synergy	Quintero Ruiz et al. (2017)
Thymol/p-cymene	Antigenotoxicity	Synergy	Quintero Ruiz et al. (2017)
Carvacrol/thymol	Against pathogenic bacteria	Synergy	Kissels et al. (2017)
Carvacrol/thymol	Against rhipicephalus microplus, R. sanguineus (Acari:Ixodidae)	Synergy	Araújo et al. (2016)
Carvacrol/thymol	Cytotoxicity	Synergy	Coccimiglio et al. (2016)
Carvacrol/1,8-cineole	Inhibition of staphylococcus aureus	Synergy	Honório et al. (2015)
Thymol/carvacrol	Antibacterial – against *Brachyspira hyodysenteriae*	Synergy	Maele et al. (2016)
Thymol/carvacrol	Against dermacentor nitens (Acari: Ixodidae)	Synergy	Novato et al. (2015)
Carvacrol/20 substances	Against culex quinquefasciatus (mosquito)	Synergy	Pavela (2015)
Borneol/20 substances	Against culex quinquefasciatus (mosquito)	Synergy	Pavela (2015)
Camphor/20 substances	Against culex quinquefasciatus (mosquito)	Synergy	Pavela (2015)
Thymol/carvacrol	Against cisplatin – induced nephrotoxicity	Synergy	EL-Sayed et al. (2015)
Carvacrol/thymol	Against culex pipiens pallens (Diptera: Culicidae)	Synergy	Ma et al. (2014)
Carvacrol/thymol	Against enterococcus faecalis	Synergy	Gutiérrez-Fernández et al. (2013)
Carvacrol/thymol	Antimicrobial	Synergy	Guarda et al. (2011)
Carvacrol/p-cymene	Antimicrobial	Synergy	Custódio et al. (2011)
Thymol/p-cymene	Antimicrobial	Synergy	Custódio et al. (2011)
Thymol/carvacrol	Against *E. coli*	Synergy	Pei et al. (2009)
Carvacrol/cymene	Against listeria monocytogenes	Synergy	Periago et al. (2004)
Carvacrol/p-cymene	Antibacterial	Synergy	Burt (2004)
Thymol/carvacrol	Antioxidant	Synergy	Vardar-Unlü et al. (2003)
Carvacrol/cymene	Antimicrobial	Synergy	Ultee et al. (2000)
Thymol/carvacrol	Antibacterial	Synergy	Didry et al. (1994)
1,8-cineole/camphor	Insecticide	Synergy	Tak and Isman (2017)
Carvacrol/1,8-cineol	Against staphylococcus aureus	Synergy	Honório et al. (2015)
1,8 cineol/terpinene-4-ol	Against botrytis cinerea	Synergy	Yu et al. (2015)
1,8 cineol/camphor	Against trichoplusia ni	Synergy	Tak et al. (2016)
1,8-cineol/carvacrol	Antibacteria	Synergy	De Sousa et al. (2012)
1,8-cineol/caryophyllene oxide	Anticholinesterase	Synergy	Savelev et al. (2003)

## Safety Issues

Carvacrol, eucalyptol and *β*-caryophyllene, *p*-cymene, *γ*-terpinene, borneol and *α*-terpineol have been approved by the Food and Drug Administration (FDA) for food use (Code for Federal Reguation: 21CFR172.515). Moreover, the European Commission Implementing Regulation (EU No 872/2012) of October 1, 2012, based on the evaluations of EFSA, included carvacrol, eucalyptol and *β*-caryophyllene, in the Union’s List of Flavorings and Source Materials (Table 6). Their use is therefore permitted, in accordance with good agricultural and manufacturing practices and without given specific restrictions. This EU list takes also into consideration the reports of the Chemical Abstracts Service (CAS), the Joint FAO/WHO Expert Committee on Food additives (JECFA) and the Council of Europe. The rest of the constituents included in the essential oil’s composition of the mixture of *Coridothymus capitatus*, *Salvia fruticosa* and *Origanum dictamnus* (Duikler et al., 2015), are also included in the same list, concerning Food supplements among others, as defined in Directive 2002/46/EC of the European Parliament and the Council, excluding food supplements for infants and young children.

**TABLE 6:** Chemical constituents of herb essential oil preparations, which are included in the Union List of Flavourings and Source Materials (Commission Implementing Regulation (EU) No 872/2012 of October 1, 2012) based on EFSA and/or JECFA evaluations.

FL-no	Substance	CAS-number	JECFA-number	CoE-number	Restrictions of use	References
04.031	Carvacrol	499–75-2	710	2055	-	EFSA
03.001	1,8-cineol	470–82-6	1,234	182	-	EFSA
01.007	β-caryophyllene	87–44-5	1,324	2,118	-	EFSA
01.002	*p*-cymene	99–87-6	1,325	620	-	EFSA
01.020	γ-terpinene	99–85-4	1,340	11,025	-	EFSA
02.016	Borneol	507–70-0	1,385	64	-	EFSA
02.014	α-terpineol	98–55-5	366	62	-	JECFA
01.008	β-myrcene	123–35-3	1,327	2,197	—	EFSA
02.085	*cis*-sabinene hydrate	546–79-2	441	10,309	—	JECFA
02.085	*trans*-sabinene hydrate	546–79-2	441	10,309	—	JECFA
02.013	Linalool	78–70-6	356	61	—	JECFA
07.215	Camphor	464–49-3	1,395	140	There is maximum dose per day	EFSA
02.230	δ-terpineol	8,000–41-7	—	—	—	EFSA
02.072	Terpinen-4-ol	562–74-3	439	2,229	—	JECFA
04.006	Thymol	89–83-8	709	174	—	EFSA
—	δ-terpinyl acetate	—	—	—	—	—
16.043	Caryophyllene oxide	1,139–30-6	1,575	10,500	—	EFSA
08.014	n-hexadecanoic acid	57–10-3	115	14	—	JECFA

However, in addition to these beneficiary compounds, the extract of the three aromatic plants includes also 0.74 and 0.52% of *cis*- and *trans*-thujone respectively (Duijker et al., 2015). Alpha and beta thujone are, according to the Regulation EC No 1334/2008 of the European Parliament and of the Council of December 15, 2008, among the substances which shall not be added as such, to food or food supplements. Maximum concentrations of thujone, naturally present in flavorings and food ingredients with flavoring properties, have been introduced. According to Regulation EC No 1334/2008, “*The maximum levels shall not apply where a compound food contains no added flavourings and the only food ingredients with flavoring properties which have been added are fresh, dried or frozen herbs and spices. After consultation with the Member States and the Authority, based on data made available by the Member States and on the newest scientific information, and taking into account the use of herbs and spices and natural flavoring preparations, the Commission, if appropriate, proposes amendments to this derogation.*” EMA/HMPC (2010) reported that exposures in the range between 3 and 7 mg/day do not pose special concerns. For higher concentrations, a case-by-case benefit/risk assessment is necessary (Lachenmeier and Uebelacker, 2010; Pelkonen et al., 2013; Sotiropoulou et al., 2016). Finally, Dettling et al. (2004) showed that a single dose of 0.28 mg/kg in men (20 mg/70 kg) and of 0.24 mg/kg (17 mg/70 kg) in women provided “borderline relevance” of adverse effects, mainly related to perturbations in driving, operating machinery, etc.

## A Case Study of the Use of Plant Extracts as a Pharmaceutical Product: Lessons Learned

The idea of using herb extracts for the treatment of upper respiratory infections was raised several years ago, when epidemiological observations revealed that people who consumed certain herbs infusions had low rates of respiratory infections and rarely suffered from common colds or influenza infections. The efficacy of the selected herbs was attributed to their antioxidant properties (Lionis et al., 1998). After years of *in vitro* and *in vivo* research, an essential oil combination was developed, based on the extracts of the three plants discussed in the present review (*Coridοthymus capitatus*, *Salvia fruticosa* and *Origanum dictamnus*) in extra-virgin olive oil (Duijker et al., 2015); we showed that this combination exerted a synergistic effect against viral upper respiratory tract infections, including influenza (Anastasaki et al., 2017). Moreover, an *in vitro* study revealed that this combination exhibited a remarkable direct antiviral activity against influenza A/H1N1 viral strains, influenza B and human rhinovirus 14 (HRV14), related to a defective trafficking of influenza A Nucleoprotein (NP) (Tseliou et al., 2019). In this respect, the “one drug, one target, one disease” approach (Zhou et al., 2016) was “violated.” Indeed, the combinatorial use of herbal preparations resulted in an synergistic effect, beyond the reported properties of each plant. Hence, here we address the case of the development and commercialization of a product, containing this essential oils’ combination.

**FIGURE 3:**
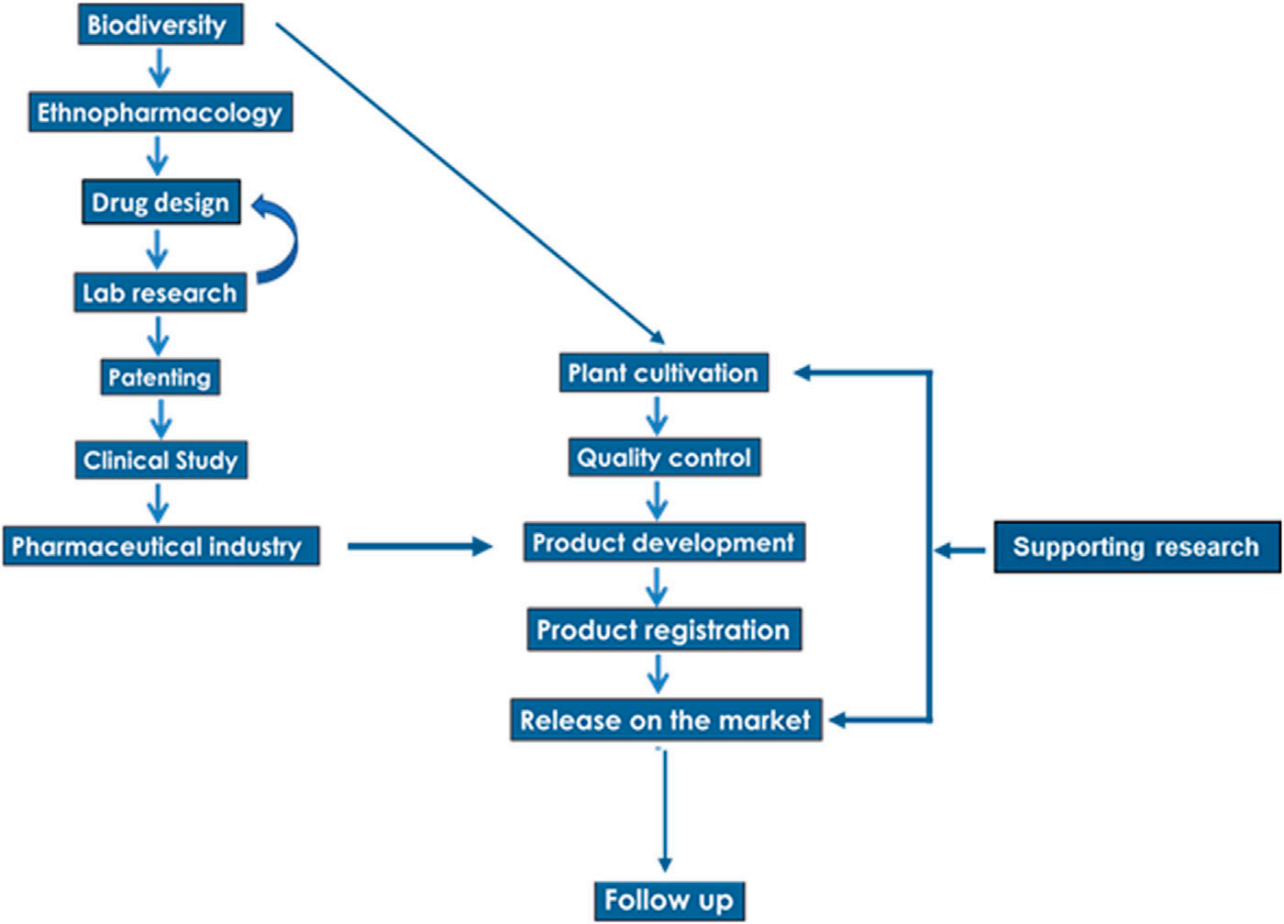
The flow-chart of a translational chain development.

The scheme of the translational chain, from the step of biodiversity to the development of a commercial product, is presented in Figure 3. The first edge of the chain, leading from a plant extract to a final product, relies on the choice of plants, as well as the choice of secondary metabolites, whose biological activity is expected to ensure the desired health benefit (*drug design*). For example, the preparation containing the essential oils of *Thymbra capitata* (L.) Cav., *Salvia fruticosa* Mill. and *Origanum dictamnus* L. avoided plant extracts rich in alkaloids, as preliminary (Lionis et al., 1988) and clinical (Duijker et al., 2015) or laboratory evidence (Tseliou et al., 2019) documented a potent health benefit of the preparation.

Another part of the continuum is related to the implementation of a *clinical study*, which is one of the main obstacles in the translational process. In the presented case, it was valuable not only for the demonstration of an antiviral action, but also for the evaluation of the amelioration of the symptoms of the disease, due to supplementary actions of the substances involved in the combination of essential oils.

The supply of raw material is an important part of the translational chain. It is well known that several restrictions rule the natural collections (harvests) and trade of herbs and spices, especially within the framework of the EU environmental policy, as well as within the framework of the United Nations Convention on Biological Diversity. For example, 232 medicinal and aromatic plant species are listed on Appendix II of CITES, which regulates the international trade in endangered species. Worldwide, the increased needs for medicinal plants, in combination with the strengthening of the regulatory framework for their collection and trade, have caused supply constraints of medicinal plants of high ethnopharmacological interest, which introduced a serious constrain in the development and commercialization of plant-derived products (Amirkia and Heinrich, 2014). In the presented case, the plants are *cultivated* under controlled and monitored conditions, while local farmers have an important income from this agricultural activity. This activity constitutes a viable developmental axis for the local communities.

Recently, a new tool based on Ecological Niche Modeling has been developed, in order to support local farmers in the decision-making process, concerning the suitability of the area where their land is located, for cultivation of medicinal taxa of high ethnopharmacological interest (Bariotakis et al., 2019). A web-based, easy-to-use application was created in the framework of precision agriculture, where the predicted suitability scores for each area of interest can be made accessible to anyone, by the use of its GPS coordinates. So, in our case, the raw material is produced from organic farming, with Global GAP and precision agriculture.

Finally, it should be clear that the continuum in plant-based drug development, is not terminated at the step of release on the market. A *follow-up* of the final product, after its market release and additional supporting research is necessary. In the example case presented here, a follow up was made through a pragmatic prospective observational study (Anastasaki et al., 2017). In our opinion, this “supporting research” step is necessary as, in many cases, there are further research queries which need clarification even after the pharmaceutical evidence of an essential oil combination. In the example case, the effectiveness of the combination of essential oils in humans was documented (Duijker et al., 2015), but the mode of biological action was not understood. Limited *in vitro* data was available with ongoing research (Tseliou et al., 2019), providing a possible mechanism, at a cellular level.

In summary, two main lessons emerged from the development of a new pharmaceutical product: The first is resumed in the words “mind the gap,” a well-known phrase from the London underground, as it reveals the necessity of the bridging between successional steps, or successional links of the translational chain. The second concerns the time lag in bridging some successional steps. Indeed, in practice, many subsequent successive steps are fulfilled before others, imposing several loops in the development of the final preparation, which should be treated and resolved accordingly.

However, in spite of the successful use of the three plants, further research is required, in order to decipher additional multi-target action(s), in view of supplementary beneficial effects, which need to be investigated/screened not only *in vitro* but also in preclinical and clinical studies in the context of Evidence Based Medicine (Stavrou et al., 2013). The results of this screening are expected to clarify whether the ethnopharmacological gap, between reported traditional uses in ethnobotanical studies and the tested properties, is due to a noise of data collection in ethnobotanical practice or reflects underline biological activities, which should be incorporated in the formal therapeutic practice. Moreover, there are no preclinical or clinical evidence about the exposure of sensitive groups (i.e., pregnant women, children, etc.).

A logical subsequent step might be the development a drug, instead of a dietary supplement. A number of items are required for this shift, especially: 1) the repetition of phase I/II trial, with a greater number of participants, together with a detailed pharmacokinetic study of the major active compounds; 2) the performance of a phased III trial. Phase III studies, undertaken in large numbers of patients, often in multiple centers, assess real outcomes in a variety of patients, approximating the global population of patients, who will receive the drug. Their aim is to compare new treatments with existing ones and to demonstrate long-term safety and tolerance (Hobbs and McCarthy, 2009).

Finally, taking into consideration reported variability of the plants’ chemical fingerprint, at least in two of the three native Cretan herbs, which have been used for the development of the active extract (see Karousou et al., 2000; Karousou et al., 2005), it becomes clear that the biological variability in nature does not conform to the requirements for stability in the composition required by market regulations. Thus, further study is suggested concerning the interaction of environmental, chemical, genetic and epigenetic factors, for the quality assurance process. Last but not least, further studies on the cultivation and storage of the above species are required for the standardization and quality control measures, along the whole supply chain.

## Author Contributions

EC, SP, CL contributed conception and design of the study, SP and EC wrote the first draft of the manuscript, CL, GS, MK and MB wrote sections of the manuscript. All authors contributed to manuscript revision, read and approved the submitted version.

## Conflict of Interest

SP, CL, and EC are inventors of a patent (WO2010GB01836 20,100,929) on the use of the three examined plants for combating upper respiratory tract infections.

The remaining authors declare that the research was conducted in the absence of any commercial or financial relationships that could be constructed as a potential conflict of interest.

## Funding

This work was partially supported by a grant from OLVOS Pharmaceuticals. During the peer review of this manuscript, an additional study of our group was published (Kalyvianaki K, Malamos P, Mastrodimou N, Manoura-Zonou I, Vamvoukaki R, Notas G, Malliaraki N, Moustou E, Tzardi M, Pirintsos S, Lionis C, Sourvinos G, Castanas E and Kampa M. Toxicity evaluation of an essential oil mixture from the Cretan herbs Thyme, greek sage and cretan ddittany, npj Science of Food (2020) 4:20; doi:10.1038/s41538-020-00080-1, suggesting the absence of accute or sub-chronic toxicity of the three herb preparation.
